# Global Epileptic Seizure Identification With Affinity Propagation Clustering Partition Mutual Information Using Cross-Layer Fully Connected Neural Network

**DOI:** 10.3389/fnhum.2018.00396

**Published:** 2018-10-02

**Authors:** Fengqin Wang, Hengjin Ke

**Affiliations:** ^1^Huangshi Key Laboratory of Photoelectric Technology and Materials, College of Physics and Electronics Science, Hubei Normal University, Huangshi, China; ^2^Computer School, Wuhan University, Wuhan, China

**Keywords:** affinity propagation clustering, MI, EEG, epilepsy, synchronization, pattern classification

## Abstract

A longstanding challenge in epilepsy research and practice is the need to classify synchronization patterns hidden in multivariate electroencephalography (EEG) data that is routinely superimposed with intensive noise. It is essential to select a suitable feature extraction method to achieve high recognition performance. A typical approach is to extract the mutual information (MI) between pairs of channels. This calculation, which considers the differences between the sequence pairs to build a reasonable partition, can improve the classification performance. On this basis, however, it is even more difficult to adaptively classify the synchronization patterns hidden in multivariate EEG data under circumstances of insufficient *a priori* knowledge of domain dependency, such as denoising, feature extraction on a special patient, etc. To address these problems by (1) effectively calculating the MI matrix (synchronization pattern) and (2) accurately classifying the seizure or non-seizure state, this study first accurately measures the synchronization between channel pairs in terms of affinity propagation clustering partition MI (APCPMI). The global synchronization measurement is then obtained by organizing APCPMIs of all channel pairs into a correlation matrix. Finally, a cross-layer fully connected net is designed to characterize the synchronization dynamics correlation matrices adaptively and identify seizure or non-seizure states automatically. Experiments are performed using the CHB-MIT scalp EEG dataset to evaluate the proposed approach. Seizure states are identified with an accuracy, sensitivity, and specificity of 0.9793 ± 0.002, 0.9942 ± 0.0005, and 0.9676 ± 0.003, respectively; the resulting performance is superior to those achieved by most existing methods over the same dataset. Furthermore, the approach alleviates the necessity for strictly preprocessing (denoising, removing interferences and artifacts) the EEG data using prior knowledge, which is usually required by existing approaches.

## 1. Introduction

To understand the intrinsic mechanisms of brain functions or disorders, researchers often classify synchronization patterns from multivariate electroencephalography (EEG) data and depict the interactions between different brain regions (Gysels, 2005). It is therefore unsurprising that understanding brain synchronization patterns has long been a central goal of neuroscience (Kandel et al., 2013), with respect to conditions such as epilepsy. The many applications of synchronization patterns in multivariate EEG include feature extraction (Mirowski et al., 2009), complex oscillator networks, neural computing (Cui et al., 2016), and brain disorder detection (Chen et al., 2014). Synchronization measurement of EEG represents an effective means of characterizing the underlying brain dynamics, e.g., identification and prediction of brain states. A typical example is the need to identify evolving synchronization patterns from multivariate EEG data in epilepsy research and clinical practice. The huge diversity of EEG data from different patients makes this task even more challenging.

Early studies on EEG synchronization focused on bivariate synchronous analysis, using measures such as the Pearson correlation coefficient, Spearman rank correlation, and mutual information (MI). MI is one of the most important information independence metrics (Cui et al., 2016), and it performs better than others in terms of anti-noise capability (Bonita et al., 2014). A difficult and unresolved problem in MI calculation is the determination of thresholds based on partitions. The traditional MI between pairs of EEG signals (Kumar et al., 2017; Piho and Tjahjadi, 2018) is calculated based on the probability distribution of continuous random variables. The variables are divided into partitions with the same probability, e.g., uniform distribution. However, there is no clear evidence that multivariate EEG data obey a certain probability distribution. Affinity propagation (AP) (Frey and Dueck, 2007) is a readily extensible clustering algorithm. It demonstrates significant improvements compared with other approaches (K-means, spectral clustering, and super-paramagnetic clustering) owing to the following advantages: (1) it is not necessary to specify the number of exemplars before applying the AP algorithm, and (2) comparable or better results can be obtained in far less time for large datasets (Wei et al., 2017). Thus, this paper first uses the AP algorithm to divide the EEG channel signals, and then calculates the MI accurately. In recent years, great progress has been made with respect to multivariate synchronous analysis approaches, such as complex networks, S-estimation (Carmeli et al., 2005), and correlation matrix analysis (Cui et al., 2010). Among these, S-estimation can effectively measure global synchronization, but cannot measure the synchronization details between bivariate. Complex networks can be used to obtain topological details of different variables, but has notable deficiencies in global synchronization measurements, while correlation matrix analysis has the advantages of both the former methods. In summary, the approach presented in this work first measures the synchronization using AP clustering partition MI (APCPMI) between pairs of channels. It then organizes all APCPMI values into a correlation matrix.

Classification of the patterns hidden in multivariate EEG has long been an interesting area of research with respect to brain diseases such as epilepsy. Traditional methods focus on time frequency analysis and synchronization measurements. Recently, machine learning methods have become popular. Myers et al. proposed a seizure prediction and classification algorithm for the CHB-MIT scalp EEG dataset, involving calculation of phase/amplitude lock values. It achieved a sensitivity of 0.77, a precision of 0.88, and 0.17 false positives per hour (Myers et al., 2016). To find EEG segments indicating seizures and their onset and offset points, Orosco et al. developed a patient non-specific strategy for seizure detection based on stationary wavelet transforms of EEG signals and achieved a specificity of 0.999, sensitivity of 0.875, and a false positive rate per hour of 0.9 (Orosco et al., 2016). Behnam et al. proposed a density-based real-time seizure prediction algorithm based on a trained offline seizure detection model. This method achieved an accuracy rate of 0.8656, a precision rate of 0.8653, and a recall rate of seizure prediction of 0.9727, and the false prediction rate was 0.00215 per hour using their online signal prediction algorithm (Behnam and Pourghassem, 2016). Fergus et al. proposed a new method for generalizing seizure detection across different subjects without *a priori* knowledge about the focal point of seizures using the CHB-MIT scalp EEG dataset (Fergus et al., 2016). Classification was enabled by the k-NN algorithm and achieved a sensitivity of 0.88 and a specificity of 0.88. Mirowski et al. proposed a method to classify patient-specific synchronization patterns to predict seizure onset over the Freiburg dataset (Mirowski et al., 2009). EEG synchronization was measured via cross-correlation, non-linear interdependence, dynamical entrainment, and wavelet synchrony. Spatial-temporal patterns were then extracted to support seizure onset prediction, with a sensitivity of 0.71 and zero false positives. EEG synchronization between the left and right parasagittal, and between the frontal and parietal brain regions was assessed with 4 different quantitative measures (delta power asymmetry, cross-correlation, mutual information, and transfer entropy). Their method achieved a specificity of 1.0, a sensitivity of 0.54, and an accuracy 0.81 for seizure detection with video-EEGs recordings (Zubler et al., 2017). Traditional methods focus on classifying EEG synchronization patterns in terms of linear [e.g., kappa statistics (Slooter et al., 2006) and K-means (Quyen et al., 2005)] and non-linear classifiers [e.g., support vector machine (SVM) (Gysels et al., 2005)]. EEG data are routinely non-linear and non-stationary in nature, and the synchronization patterns (if any) embedded in them are inevitably highly non-linear. This always results in poor performance for linear classifiers (Quyen et al., 2005; Slooter et al., 2006). In particular, kappa is incapable of revealing synchronization patterns in detail, and K-means is often trapped at local optima owing to its high sensitivity to noises and outliers. The SVM is applicable to non-linear problems, but it cannot find a general solution to EEG synchronization classification as (1) selection of the kernel function is problem-specific and (2) the space information among synchronization patterns is discarded. To solve this problem, non-linear adaptive pattern recognition technologies, such as deep neural network (NN) approaches, have a vital role in non-linear analysis, as they are self-adaptive and have strong fault tolerance. In contrast to previous work, this study aimed to find a solution capable of adaptive classification of non-stationary synchronization patterns to capture the intrinsic nature of seizure activities represented by the EEG. Besides obtaining better classification performance, the classifier presented here has the merits of (1) fast training speed, and (2) alleviating overfitting and enhancing generalization ability.

To tackle the challenges, we first extracted a global synchronization feature that could effectively suppress strong noise. Then we designed a cross-layer fully connected NN (CLFCNN) classifier to classify the presence or absence of an epileptic seizure. The experimental results presented in this paper were evaluated on the CHB-MIT public authorized dataset (Goldberger et al., 2010). Compared with most existing methods, the classifier proposed in this paper achieves high accuracy, sensitivity, and specificity. It can also be applied to complex science and engineering applications effectively, because not only does it not require *a priori* knowledge of subjects dependent on the problem domain, but only a single time window parameter needs to be set manually, which can greatly reduce various errors caused by improper parameter settings. The main contributions are as follows.

- For multivariate EEG data subjected to strong noise and interference, a synchronous evolution pattern feature extraction method, named APCPMI, was designed. Compared with the traditional MI calculation method, APCPMI fully considers the differences in the channel data themselves and measures the MI between EEG data pairs more accurately.
- Without *a priori* knowledge, an adaptive classifier was designed, which can effectively distinguish synchronization patterns with diversity and uncertainty. It shows excellent performance in epileptic seizure detection on the CHB-MIT dataset.
- In order to make the model more robust (without overfitting) and generalized, the proposed method was aimed at the whole sample space without sufficient *a priori* knowledge to perform the preprocessing (noise removal or interference removal) that is widely used in existing methods.

The remainder of this paper is organized as follows. The second section introduces the proposed method of CLFCNN based on an APCPMI correlation matrix. The third section proposes the case study, that is, epilepsy seizure classification. Finally, the main highlights of this paper are emphasized.

## 2. Methods

This section discusses in detail the main concepts and methods used in this work. First, the materials used in the study are described. Second, we summarize the overall design of our solution. The global synchronization measure, a correlation matrix based on APCPMI (CMAPCPMI), is discussed in detail. Finally the implementation of the classifier (CLFCNN) is discussed.

### 2.1. Materials

The CHB-MIT scalp EEG dataset, which is publicly authorized for open access, was used for this study. The dataset consisted of EEG recordings from 22 patients (5 males aged 3–22 and 17 females aged 1.5–19) with severe epilepsy caused by organic lesions, which were recorded simultaneously through 23 different channels (FP1-F7, F7-T7, T7-P7, P7-O1, FP1-F3, F3-C3, C3-P3, P3-O1, FZ-CZ, CZ-PZ, FP2-F4, F4-C4, C4-P4, P4-O2, FP2-F8, F8-T8, T8-P8, P8-O2, P7-T7, T7-FT9, FT9-FT10, FT10-T8, and T8-P8) at 256 Hz with 19 electrodes and a ground attached to the surface of the scalp. Most recordings contained multiple seizure occurrences.

This study investigated EEG recordings with the same number of channels (from 18 patients). To avoid the problems of imbalanced samples, Markov chain Monte Carlo (MCMC) (Robert and Casella, 2004) sampling was used to balance the seizure state and non-seizure state samples. The details were as follows. (1) For each epileptic seizure stage with size *S*(*seizure*), synchronization matrix counts for seizures were denoted as *count*(*seizure*) = ⌊*S*(*seizure*)/*S*(*window*)⌋, where *S*(*window*) is the size of the time window. (2) Synchronization matrix counts for non-seizure stage prior to epileptic seizure stage were denoted as $count\left(prior\right)=\left\lfloor \frac{1}{2}\times S\left(seizure\right)/S\left(window\right)\right\rfloor$. (3) Synchronization matrix counts for non-seizure stage posterior to epileptic seizure stage were denoted as *count*(*posterior*) = *count*(*seizure*)−*count*(*prior*). Cases containing the same channel numbers were used, corresponding to 18 patients, as shown in Table 1. All EEG data with epileptic seizures were divided into 753 non-overlapping time windows (segments), each of which contained 2,048 points (8 s). The non-seizure state segments (753) were then obtained by MCMC sampling.

**Table 1:** Details of the EEG records.

**No**.	**Gender**	**Age (years)**	**Seizure count**
1	F	11	7
3	F	14	7
5	F	7	5
7	F	14.5	1
8	M	3.5	5
9	F	10	1
11	F	12	3
12	F	2	27
13	F	3	10
14	F	9	4
16	F	7	7
17	F	12	2
18	F	18	6
19	F	19	2
20	F	6	8
21	F	13	4
22	F	9	3
23	F	9	2

### 2.2. Overall design

**Figure 1:**
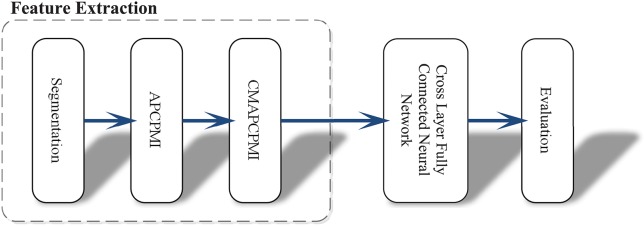
Overview of the proposed approach and its operation process. First, the raw EEG data is segmented with the same time window. Then all APCPMIs between pairs of channels are calculated and organized into a correlation matrix based on APCPMI (CMAPCPMI). Finally, the feature matrices are trained by CLFCNN and predicted as seizure or non-seizure states.

In view of the time requirements of clinical applications, this approach was intended to avoid the conventional and heavy data preprocessing tasks (denoising, removing interference and artifacts). Another obstacle to clinical applications is that existing methods rely heavily on *a priori* knowledge and require a large number of hyper-parameter settings. Figure 1 shows the whole procedure of this method in three stages: (1) synchronous feature extraction, (2) pattern classification based on CLFCNN, and (3) evaluation of performance.

### 2.3. Global synchronization measurement

#### 2.3.1. Mutual information

MI is a natural measure for selecting useful features because it expresses the uncertainty between bivariate data (Shannon, 1948; Ullman and Bart, 2004). It does not need any hypothesis or *a priori* knowledge to measure the correlation between bivariate data. Let X and Y be discrete variables, and let the corresponding probability distributions and the joint probability distribution be represented by p(X), p(Y), and p(X, Y), respectively. The entropies (H(X) and H(Y)) and the joint entropy (H(X, Y)) are defined as follows:

(1) $$\begin{array}{l} H(X)=-\underset{x\in X}{\sum^{\text{​}}}p(x)log_{2}p(x),\\ \;H(Y)=-\underset{y\in Y}{\sum^{\text{​}}}p(y)log_{2}p(y),\\ H(X,Y)=-\underset{x\in X,y\in Y}{\sum^{\text{​}}}p(xy)log_{2}p(xy) \end{array}$$

The MI is calculated as below:

(2) $$\begin{array}{r} I\left(X,Y\right)=H\left(X\right)+H\left(Y\right)-H\left(X,Y\right) \end{array}$$

**Figure 2:**
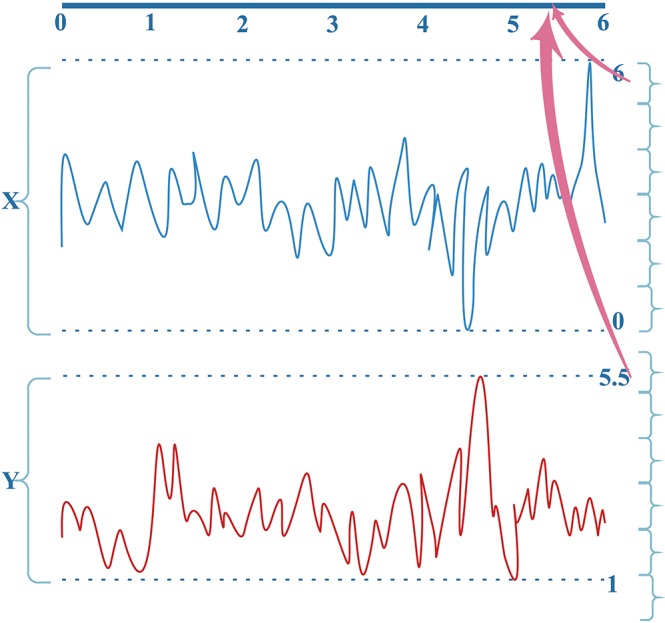
The conventional computation of MI. X(X ⊆ [0,6]) and Y (Y ⊆ [1, 5.5]) are the bivariate signals that denote the pairs of channels. Different colors indicate different signals X and Y. See text for details.

**Figure 3:**
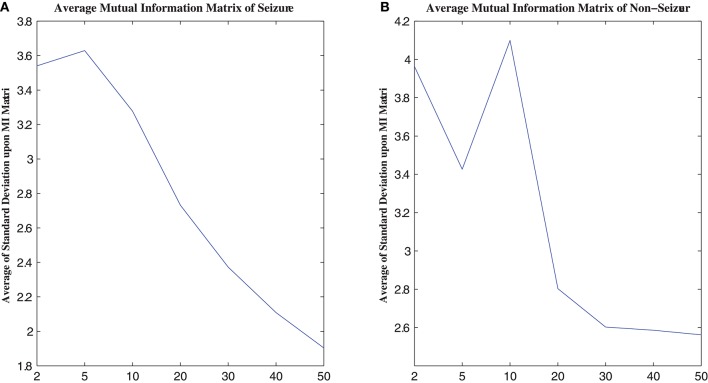
The relation between MI calculation and partition number. **(A)** is the average MI matrix for all seizure states, and **(B)** is the average MI matrix for non-seizure states. See text for details.

MI computation is strongly dependent on data partitioning. In this method, for each segment of a seizure or non-seizure state, the MI matrix (23 × 23) was calculated in terms of different partition numbers, which are divided according to the uniform distribution. Figure 2 illustrates the traditional method of computing MI. Amplitude values belonging to the same partition will fall into the corresponding partition; self-entropy and joint entropy are then calculated using Equation 1 (denoted as arrows in Figure 2). Finally, the MI is calculated using Equation 2. The average standard deviation values of all MI matrices for different partition numbers are illustrated in Figure 3. This shows that the MI calculation is strongly dependent on data partitioning. In view of this partitioning problem, an adaptive partitioning algorithm is essential; such an algorithm is briefly introduced in the following sections 2.3.2 and 2.3.3.

As shown in Figure 3, the standard deviations of the non-seizure state MI matrix were greater than those of the seizure state MI matrix.

#### 2.3.2. AP clustering algorithm

AP clustering is an algorithm based on information transferred between data points. Compared with classical clustering analysis algorithms, it does not need to determine the number of clusters before running, and it iterates through competitive clustering centers for each sample point to achieve the best clustering performance.

The input of the AP algorithm is the similarity between the sample data *s*[*i, j*](*i, j* = 1, 2, …, *N*), e.g., Euclidean distance, Kullback-Leibler divergence, or cosine distance. This paper uses the Euclidean distance to represent the element values in the similarity matrix S. The element on the diagonal line of S is a reference matrix P, which indicates the probability that each sample point is selected as a partition center. The AP algorithm iterates through the sample data to construct the responsibilities matrix R(i, k) and the availability matrix A(i, k) until the appropriate partition center *x*_*k*_ is found. The iterative formulas are as follows.

(3) $$\begin{array}{r} \begin{array}{cc} R\left(i,k\right) & \leftarrow S\left(i,k\right)-\underset{k^{\prime }s.t.k^{\prime }\neq k}{\max}\left\{A\left(i,k^{\prime }\right)+S\left(i,k^{\prime }\right)\right\},\\ A\left(i,k\right) & \leftarrow min\left\{0,R\left(k,k\right)+\underset{i^{\prime }s.t.i^{\prime }\notin \left\{i,k\right\}}{\sum }max\left\{0,R\left(i^{\prime },k\right)\right\}\right\} \end{array} \end{array}$$

AP clustering uses the two equations in Equation 3 alternately. For a detailed implementation, refer to Frey and Dueck (2007). Compared with the K-means approach, the main advantages are as follows: (1) no manual initial partition center is required; (2) the partition center is a real existing data sample instead of a virtual new one; (3) it is insensitive to the initial value; and (4) the squared error of the result is smaller (Frey and Dueck, 2007).

#### 2.3.3. AP cluster partition mutual information

The calculation of APCPMI involves three stages. First, the bivariate signals X and Y are ordered (ascending) to speed up the convergence of AP clustering. Second, the variables are partitioned, respectively, with the AP clustering algorithm to obtain the coordinates of the maximum ($Z_{max}^{i}$) and minimum values ($Z_{min}^{i}$) for each partition *i*. The partition center *C*_*i*_ and corresponding partition radius *R*_*i*_ can be calculated as follows:

(4) $$\begin{array}{r} C_{i}=\frac{Z_{max}^{i}+Z_{min}^{i}}{2},R_{i}=\frac{\left|Z_{max}^{i}-Z_{min}^{i}\right|}{2},s.t.\;Z\in X,Y. \end{array}$$

where *Z* denotes the coordinates of point in one partition.

The partition center and corresponding partition radius are from the partition *P*_*i*_. Given two partitions *P*_*i*_ and *P*_*j*_, the dividing point should be the following:

(5) $$\begin{array}{r} D\left(i,j\right)=\frac{\left(C_{j}-R_{j}\right)-\left(C_{i}+R_{i}\right)}{2},\;s.t.j>i \end{array}$$

It is very likely that different partition numbers (*N*(*X*) and *N*(*Y*), *N*(*X*)≠*N*(*Y*)) between the bivariate signals can be generated. In view of this, this study merges those closer partitions with larger partition numbers until the bivariate signals contain the same partition number.

Finally, the APCPMI is calculated in terms of the partitions. The calculation details are summarized in algorithm 1.

**Algorithm 1:**
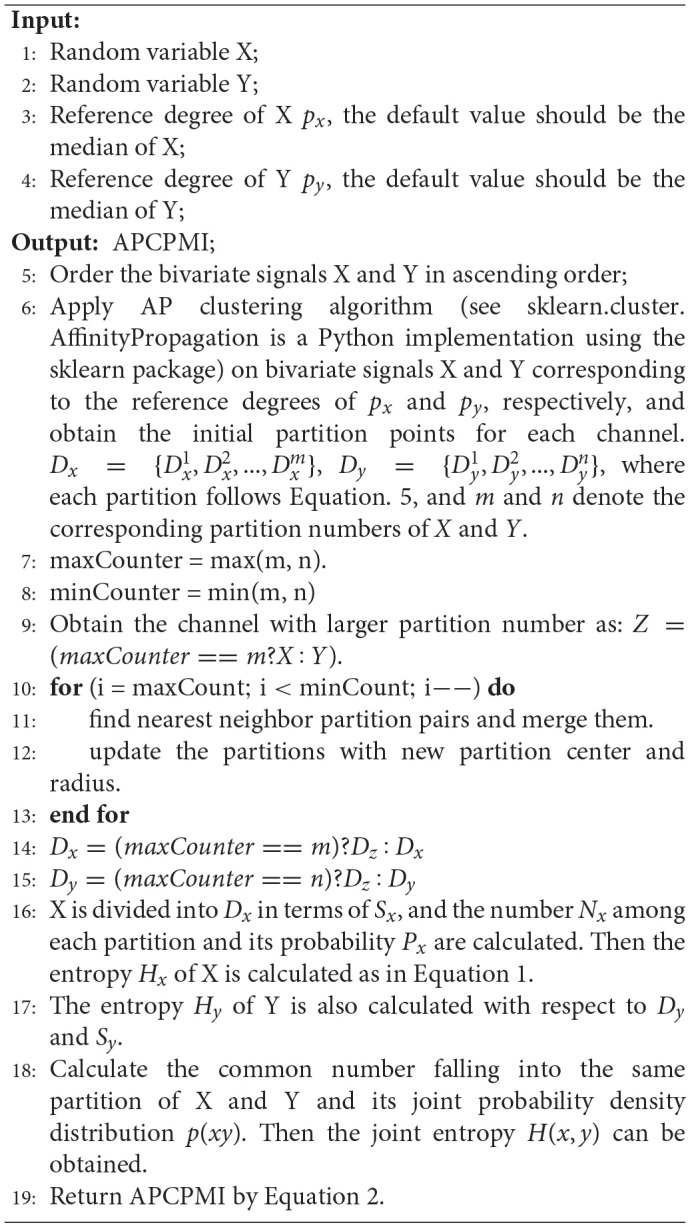
**APCPMI calculation**.

APCPMI can be used to measure the linear and non-linear synchronization between pairs of channels. In contrast to the traditional approach, it considers the differences in signal data themselves to build reasonable partitions to accurately calculate the MI.

#### 2.3.4. Correlation matrix based on APCPMI

To quantify the global synchronization of multivariate EEG, the APCPMIs are extended to form a correlation matrix based on APCPMI, named CMAPCPMI. Each element of the matrix (APCPMI) represents the synchronization measurement between the corresponding channels. The definition is as follows (MI is used in the equation for simplicity):

(6) $$CMAPCPMI=\left[\begin{array}{cccc} MI_{11} & MI_{12} & \cdots \; & MI_{1n}\\ MI_{21} & MI_{22} & \cdots \; & MI_{2n}\\ \vdots & \vdots & \ddots & \vdots \\ MI_{n1} & MI_{n2} & \cdots \; & MI_{nn} \end{array}\right]$$

where the index at each element denotes the channel index. The max index n in this study is 23.

### 2.4. Cross-layer fully connected neural network

The classifier is used to identify the EEG synchronization pattern (CMAPCPMI) for identification of epileptic seizure states. In this section, the design principle of the classifier is discussed. The overall architecture is then given. Finally, implementation details including training and test processes are proposed.

#### 2.4.1. Design principle

**Figure 4:**
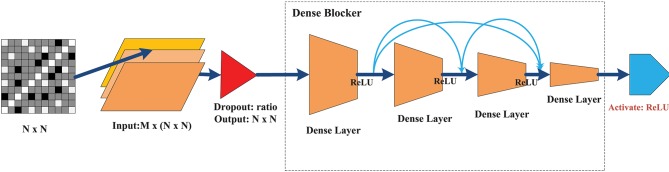
The architecture of the CLFCNN. The merge layer is ignored because there is no special parameter for this type of layer. The final activation layer with different color outputs the classification result (seizure or non-seizure). See text for details.

The goal of this classifier was to achieve a level of performance similar to that of a deep NN with minimal hidden layers to substantially reduce training time. Figure 4 illustrates the overall design architecture of a cross-layer fully connected NN. It starts with a dropout layer, followed by four layers of dense blocks forward-connected with each other (the front layer is connected to all the subsequent layers), and a final Rectified linear unit (ReLU) activation output of the seizure states. The main design principles are as follows.

- Dropout: In deep NN, dropout is used to overcome the overfitting problem. The main idea is to discard some neurons at random. When the dropout technology was proposed, Srivastava gave the ideal dropout rates as 0.2 for the input layer and 0.5 for the hidden layer (Srivastava et al., 2014). After fine-tuning this parameter, the dropout rate of the input layer was set to 0.1, while the hidden layer did not require a dropout ratio at all.
- No need for a convolution layer or pooling layer: The convolutional and pooling layer have been very successful in reducing model parameters. However, they cause significant information loss when dealing with global synchronization patterns, since the latter have low spatial resolution. Unlike images or videos, the data elements in the feature matrix exhibit little continuity.
- Merge layer: The merge layer accepts connections from all front layers and merges them in sequence with respect to the layer number. Then, each layer is mapped to the next fully connected layer, whose connection weight is fixed at 1, while bias is fixed at 0.
- Dense block: To ensure that the network stays feed-forward, the features of an earlier layer are fed as inputs into a later layer, so that the dense blockers are arranged as a forward dense net. If there are *L* layers of fully connected layers, except between two adjacent layers from the front, all layers have connections to all subsequent layers, leading to a total of $\frac{L\left(L+1\right)}{2}$ connections. The network structure is borrowed from the DenseNet (Huang et al., 2017), which can (1) alleviate the gradient vanishing problem, (2) enhance feature propagation, (3) be more conducive to feature reuse, and (4) further reduce the parameters. The main differences between the method of Huang et al. (2017) and that used here are as follows. (1) We removed the conventional and pooling layers because of non-stationary synchronous patterns. This was mainly because different channels have various synchronization patterns that can be strengthened or weakened, and convolution or pooling is likely to lose this kind of change and lead to loss of classification performance. (2) We set the merge layer to accept and merge all front layers to avoid the gradient vanishing of the front layers, while enhancing the weight propagation of the cross layer.

#### 2.4.2. Design structure of CLFCNN

The implementation details of CLFCNN are as follows: (1) the optimization uses a random batch gradient descent algorithm with batch size of 50; (2) the learning rate is set to 0.01; (3) the objective function is the mean square error; and (4) the activation function is the ReLU function. The global synchronization patterns are fed into the CLFCNN as input and its training process includes the following.

1. The forward propagation algorithm uses the outputs, weights, and bias of the prior layer as the independent variables of the current activation function. Assuming that the current layer is *L*(*i*), besides the input from *L*(*i*−1) level, there are input connections from all the connected layers before *L*(*i*−1). The output of *L*(*i*) is as follows:
  (7) $$\begin{array}{r} O_{L\left(i\right)}=\delta_{L\left(i\right)}\left\{\overset{i-1}{\underset{j=1}{\sum }}\delta_{L\left(j\right)}\left(\omega_{L\left(j\right)}x+b_{L\left(j\right)}\right)\right\} \end{array}$$
  where δ is the activation function, ω is the weight, and b is the bias.
2. The residuals of each iteration are calculated.
3. To calculate the residuals between the output and the target, the Back Propagation (BP) algorithm is utilized to back-propagate the residuals using the chain rule. This is successful because for each weight connection, there is a unique path from the output to the current connection weight.
4. After training the classifier, the trained classification model is obtained. Then test data are fed into the model, which is used to predict and classify the epileptic seizures.

#### 2.4.3. Implementation details for CLFCNN

This section describes the implementation details of the classifier. The main process is to first shuffle the whole sample space with a initial seed of 7, and divide the data into training set, validation set, and test set, which account for 0.64, 0.16, and 0.2, respectively. The training performance of the classifier is then evaluated using a five-fold cross-validation algorithm on the training set and validation set, and the final performance is reported by the test set.

##### 2.4.3.1. Model summary

Table 2 summarizes the model parameters of the proposed classifier (the last column in the table shows the number of parameters of the current layer). Although the total number of parameters (55252) of the classifier was less than that of the current mainstream deep learning framework, the classification performance of the classifier was very challenging (see section 3).

**Table 2:** The model parameter setting of the classifier.

**Inputs**	**Outputs**	**Shapes**	**Parameter**
Dropout1 (Dropout)	FC1(FC)	[None 529]	0
FC1(FC)	FC2(FC)		42,400
FC1(FC)	FC3(FC)		10,600
FC1(FC)	FC4(FC)		530
FC2(FC)	FC3(FC)	[None 80]	1620
FC2(FC)	FC4(FC)		81
FC3(FC)	FC4(FC)	[None 20]	21
FC4(FC)	Activation	[None 1]	0

*The output format of the current layer and next layer (output layer) and the number of connection parameters. The merge layer and activation layer between the hidden layer are omitted because of its zero parameter. In this work, there are two merge layers. The first is before the third layer, which accepts the outputs of the first and the second. The second is in front of the fourth layer*.

##### 2.4.3.2. Training process

In each training phase, a five-fold cross validation algorithm was used to evaluate the performance. A small batch (batch size of 50) momentum (0.9) gradient descent method was utilized to train the model, as described by Krizhevsky et al. (2012). The training is regularized by weight decay (2e-4) and dropout (0.1). The update rule for weight follows Equation 8 (Krizhevsky et al., 2012):

(8) $$\begin{array}{l} v_{i+1}\;\leftarrow 0.9\cdot v_{i}-0.0002\cdot \epsilon \cdot \omega_{i}-\epsilon {\langle \frac{\partial L}{\partial \omega }{|}_{\omega_{i}}\rangle }_{D_{i}},\\ \omega_{i+1}\leftarrow \omega_{i}+v_{i+1} \end{array}$$

where *i* is the iteration index, *v* is the momentum variable, ϵ is the learning rate, and ${\langle \frac{\partial L}{\partial \omega }{|}_{\omega_{i}}\rangle }_{D_{i}}$ is the average over the *i*th batch *D*_*i*_ of the derivative of the objective with respect to ω, evaluated at ω_*i*_.

##### 2.4.3.3. Test process

In the test phase, given the trained classifier, the raw EEG data were classified as follows. First, the EEG data were divided into the same time window as 2048, and the feature matrix (see section 2.3.3) of each window was computed. The CLFCNN classifies all sequential global synchronization patterns. The process does not need any intervention from doctors or experts. In this way, the manpower and material and financial resources required for clinical applications can be greatly reduced.

##### 2.4.3.4. Avoidance of overfitting

We used “early stopping” and “dropout” strategies to avoid overfitting of the model. In these approaches, the training accuracy is monitored continuously until it stops ascending. The iteration of training will then stop on completion of the current epoch. Taking our experiments, for example, the number of epochs was initially set to 300 while the iteration stopped at the 91st epoch. The other strategy, dropout, temporarily drops randomly selected units together with their connections from the NNs during training. The central idea of dropout is to take a large model that overfits easily and repeatedly sample and train smaller sub-models from it. This prevents units from co-adapting too much in training. At the test stage, it can approximate the effect of averaging the predictions of all these sub-models by simply using a single unthinned model that has smaller weights; thus, overfitting can be prevented in a simple manner at the cost of doubling the training time (Srivastava et al., 2014).

## 3. Experimental results and discussions

Experiments were performed to evaluate the performance of the proposed method. The testbed was a desktop with Intel i7 CPU (3.33 GHz) and 24 GB memory running 64-bit Windows 7. The experiments involved both offline training and online classification.

**Off-ine Training:** This procedure included (1) calculation of all CMAPCPMIs (the default reference degree was used to calculate APCPMI; see algorithm 1) and (2) training the NN models. With a time window of 2,048, the 10 threads simultaneously computed the AP clustering algorithm on each channel, which could be reused (each channel needed only one cluster calculation). It took about 50 seconds to calculate one synchronization matrix in JDK1.8. In summary, it took about 21.5 h to calculate all 1,406 of the global synchronization measures (753 epileptic seizures, 753 epileptic non-seizure states). The second step could output the model in 1 min.

**Online Classification:** This procedure included (1) calculation of one global synchronization in 50 s and (2) loading the trained model to perform classification, which took less than 0.01 s.

### 3.1. Evaluation of classification performance

To evaluate the performance of the classifier, 10 iterations were fulfilled. Each iteration included one whole training stage (the five-fold cross-validation method was adopted to evaluate the training performance) and a test stage. At each iteration, the feature matrices were shuffled and divided into five folds: four folds were used as training data, the remaining fold was used as validation data. The final result was the average performance from the test set of all 10 iterations.

As for seizure state identification in this study, it can be deemed as a classification problem in which the classifier is aimed to classify different seizure states (seizure state for positive, or non-seizure state for negative) presented by EEG segments and so the essence of the classifier is to construct a mapping from the feature space to class labels or seizure states here. The classifier performance is evaluated by implementing the trained classifier on the test set and comparing the predicted and actual labels. In order to clearly understand the evaluation criteria, we first introduce the confusion matrix. There are always some instances correctly classified while others incorrectly. The confusion matrix contains the most information for measuring the association between prediction and reality. On the main diagonal of the confusion matrix the number of positive cases (TP) correctly classified and the number of negative cases (TN) correctly classified and the minor diagonal reports the number of negative cases (FN) wrongly classified and the number of positive cased (FP) wrongly classified, respectively. Then, the actual positive number and the actual negative number are (TP +FN) and (FP+TN), respectively. In order to compare the performance of different classifiers more conveniently, some commonly used measures including accuracy (Equation 11), sensitivity (also known as recall in Equation 9) and specificity (Equation 10) et al., which are derived from the confusion matrix to capture information in a single scalar metric. Classification accuracy is typically taken to mean the degree to which the derived classification agrees with reality or conforms to the ‘truth.' The most common way to express the accuracy of classification is by a statement of the percentage of the seizure/non-seizure state that has been correctly classified when compared with reference data or “ground truth” calibrated by experts. Sensitivity measures the proportion of actual positives that are correctly identified as such (e.g., the percentage of seizure states who are correctly identified as seizure states). Therefore, it quantifies the avoiding of false negatives, and specificity does the same for false positives. In this study, a perfect classifier would be described as 1.0 sensitivity, meaning all seizure states are correctly identified as seizure states, and 1.0 specificity, meaning no non-seizure states are incorrectly identified as seizure. The classifier is doing a better job in correctly predicting the positives than predicting the negatives when the greater sensitivity is obtained, vice versa. The precision (also called positive predictive value in Equation 12) for a class is the number of true positives (i.e., the number of items correctly labeled as seizure state) divided by the total number of elements labeled as belonging to the positive class (seizure state). A precision score of 1.0 for seizure state means that every item labeled as belonging to seizure state does indeed belong to seizure state (but says nothing about the number of items from seizure state that are not labeled correctly). Often, there is an inverse relationship between precision and sensitivity (recall), where it is possible to increase one at the cost of reducing the other. Precision and sensitivity scores are not discussed in isolation. Instead, either values for one measure are compared for a fixed level at the other measure [e.g., precision (0.8) at a sensitivity level of 0.75)] or both are combined into a single measure. An example of measures that combine precision and sensitivity is the F-measure (the weighted harmonic mean of precision and sensitivity in Equation 14) and another example that combines sensitivity and specificity is the G-mean (Equation 13).

Let *N*_*TP*_, *N*_*FP*_, *N*_*TN*_, and *N*_*FN*_, respectively, denote the number of test dataset that the classifier has determined as true positive (TP), false positive (FP), true negative (TN), and false negative (FN) cases. A 2 × 2 confusion matrix *TPFPTNFN* can be formed from the above values (Fatourechi et al., 2008; Elyasigomari et al., 2015). In order to quantify classification performance, we used sensitivity, specificity , accuracy , precision, G-mean and F-Measure to report classification performance (Fatourechi et al., 2008; He and Garcia, 2009; Jamal et al., 2014; Mumtaz et al., 2017)^1^:

(9) $$\begin{array}{rcl} sensitivity & = & \frac{TP}{TP+FN} \end{array}$$

(10) $$\begin{array}{rcl} specificity & = & \frac{TN}{TN+FP} \end{array}$$

(11) $$\begin{array}{rcl} accuracy & = & \frac{TP+TN}{TP+FN+TN+FP} \end{array}$$

(12) $$\begin{array}{rcl} precision & = & \frac{TP}{TP+FP} \end{array}$$

where the sensitivity and specificity indicate the ratios of correctly identifying epileptic seizure and non-seizure states, respectively, and the accuracy of a classification model illustrates the percentage of correctly classified cases and non-cases among all the samples in test set. The precision calculates the proportion of all correctly identified seizure states from all that were actually classified.

The above indicators could not measure the balance performance of the classifier. For example, 100 samples, including 99 positive and 1 negative, would be reported with 0.99 high performance even if all samples were classified as positive. The G-mean and F-Measure were used to synthetically consider different performance indicators to address this problem(Fatourechi et al., 2008; He and Garcia, 2009). G-mean evaluates the degree of inductive bias in terms of a ratio of positive accuracy and negative accuracy:

(13) $$\begin{array}{r} G-mean=\sqrt{\left(sensitivity\;\times \;specificity\right)} \end{array}$$

F-Measure metric combines precision and sensitivity as a measure in terms of a ratio of the weighted importance on either sensitivity or precision as determined by the β (β = 1 in this study) coefficient:

(14) $$\begin{array}{r} F-Measure=\left(1+\beta^{2}\right)\times \frac{precision\times sensitivity}{\beta \times precision+sensitivity} \end{array}$$

**Figure 5:**
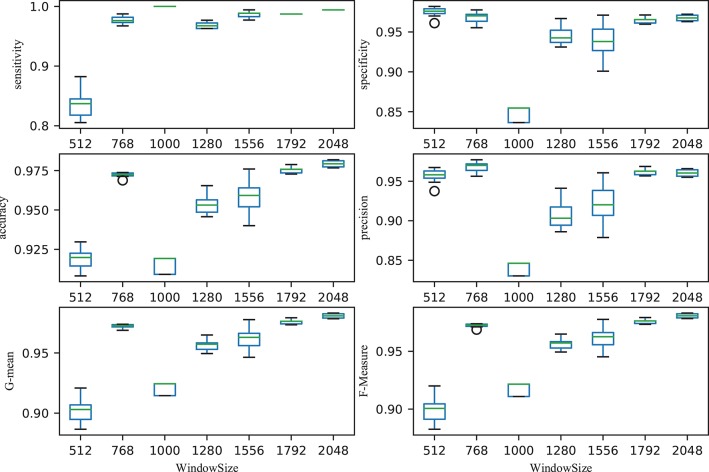
Relationship between performance (accuracy, sensitivity, specificity, precision, G-mean, and F-Measure) and window size. After ordering the data in descending order, six points were then calculated: lower (first) quartile Q1, median (second quartile) Q2, upper (third) quartile Q3, interquartile range IQR = Q3 − Q1 , lower 1.5*IQR whisker, and upper 1.5*IQR whisker. Outliers outside the lower whisker and upper whisker were plotted as a circle (see https://en.wikipedia.org/wiki/Box_plot).

The size of time window greatly affected the performance of the classifier. Figure 5 illustrated the performance with different time windows. With an increasing time window, the overall total trend was one of growth, with the exception of the time window at 1,000 (all the performance indicators were almost the lowest). With a time window of 2,048, the performance reached its peak. That is, the classification performance was reported as 0.9793 ± 0.002 accuracy, 0.9942 ± 0.0005 sensitivity, 0.9676 ± 0.003 specificity, 0.9605 ± 0.0044 precision, 0.9808 ± 0.002 G-mean and 0.9807 ± 0.002 F-Measure, respectively. The first value is average value and the second value is the corresponding standard deviation. The small standard deviations indicated the stability of the classifier.

**Figure 6:**
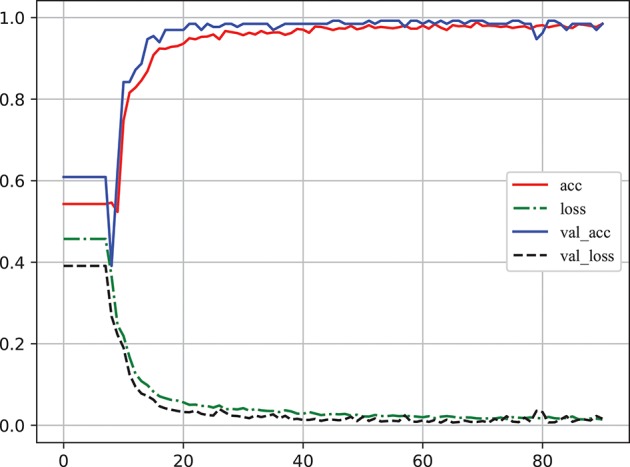
The learning curve of classifier to log accuracy and loss rates in the training stage (Video S1). *acc* and *loss* indicate the accuracy and error in training, respectively; *val*_*acc* and *val*_*loss* indicate the accuracy and error in validation, respectively. The X-axis denotes epoch [An epoch is an iteration over the entire training data provided (see https://keras.io/models/model/)]. The Y-axis denotes accuracy and loss measure in one training iteration.

Learning curve plot is widely used in machine learning to check if the model is overfitting or not. It denotes the performance (the error rate or accuracy of the learning system) changing with the number of training examples used for learning or the number of iterations used in optimizing the system model parameters (Sammut and Webb, 2010). Figure 6 illustrates the accuracy and loss metrics in the training and validation stages of one iteration which is Here, The X-axis denotes epoch (An epoch is an iteration over the entire training data provided (see https://keras.io/models/model/)). The Y-axis denotes accuracy and loss measure in one training iteration. *acc* and *loss* indicate the accuracy and error in training, respectively; and *val*_*acc* and *val*_*loss* indicate the accuracy and error in validation, respectively. It is clear that overfitting did not occur in this case as: (1) *acc* and *val*_*acc* reached high values at the same time; (2) there was no significant difference between *acc* and *val*_*acc* for any epoch; and (3) there was an excellent generalization performance, as proved by the test set.

**Figure 7:**
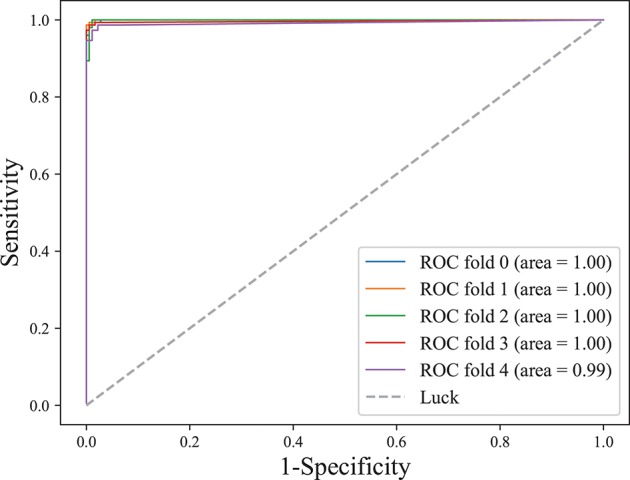
ROC curve on lightweight CLFCNN. The curve was generated from every fold of five-fold cross-validation for the CLFCNN model. See text for details.

The area under the receiver operating characteristic (ROC) curve, denoted as AUC, is widely used to measure the performance of supervised classification rules. A good performance is indicated by a convex ROC curve, which lies in the upper left triangle of the square (luck line in Figure 7, which is the diagonal reference line to represent the classification performance of random guess with AUC of 0.5) (Hand and Till, 2001). Figure 7 illustrates the classification performance (five-fold cross-validation at the training stage) for this work. The convex ROC curve indicated superior performance in classifying epileptic seizure state and non-seizures. A very large AUC with value 1 further confirmed the superior performance of the classifier.

Table 3 presents a comparison between the proposed approach and state-of-the-art methods, including those with intelligent algorithms operating on the public dataset CHB-MIT. The classifier achieved the best sensitivity and accuracy, while its specificity was only slightly worse than those of the Linear Discriminant Analysis (LDA) and NN reported in Orosco et al. (2016). Nevertheless, sensitivity was a much more critical indicator as it denoted whether seizures could be correctly detected.

**Table 3:** Performance Comparison.

**Author year**	**Classifier**	**Sensitivity**	**Specificity**	**Accuracy**	**PK**
Fergus et al. (2016)	k-NN	0.88	0.88	0.93	Y
Nasehi and Pourghassem (2013)	IPSONN	0.98	–	–	Y
Behnam and Pourghassem (2016)	MLP, Bayesian	0.8653	0.9727	0.8656	Y
Orosco et al. (2016)	LDA, NN	0.875	**0.999**	–	Y
Our approach	CLFCNN	**0.9942**	0.9676	**0.9793**	**N**

*sensitivity and specificity describe the rates of correctly detecting seizure and non-seizures states, respectively. Accuracy denotes the accuracy of classification. PK (a priori knowledge) indicates whether the corresponding approach depends on a priori knowledge. The bold values present the best performance index corresponding to the column*.

### 3.2. Discussion

#### 3.2.1. A priori knowledge

Classifiers for seizure detector have generally relied on *a priori* knowledge (Nasehi and Pourghassem, 2013; Behnam and Pourghassem, 2016; Fergus et al., 2016; Orosco et al., 2016) or patient-related data (Nasehi and Pourghassem, 2013). This results in poor generalization ability of the classifier, since researchers train and test on the same patient, or apply the classifier to different patients with specific feature extraction rules. Here, by contrast, based on all patients' samples, a general EEG classification model was established to detect epileptic seizures in different subjects. Under high noise conditions, it was important to find the synchronization pattern of multivariate EEG data and accurately classify it without sufficient *a priori* knowledge. This ability could greatly assist research into epileptic brain dysfunction.

Furthermore, the traditional classifier mostly relied on the time, frequency, and space analysis (Mirowski et al., 2009) of EEG signals. The frequency bands of different patients were often quite different. Obtaining appropriate frequency bands had become a challenging research problem, which made it difficult to classify different frequency bands from different patients. Methods aimed at solving this problem included the extraction of frequency band components based on a Bayesian framework (Suk and Lee, 2013). However, the extraction of appropriate frequency bands required complex analysis of a large number of epochs. Meanwhile, the time window had to be long enough to avoid losing useful frequency information. For example, Mirowski et al. had to apply several minutes of EEG (12–60 frames) to obtain the appropriate frequency band information, but the synchronization measure needed only a 5-s time window (Mirowski et al., 2009).

#### 3.2.2. Advantages of the proposed classifier

The latest NNs are highly suited to EEG classification as they have the following properties. (1) Non-linearity: a NN consisting of interacting neurons (linear or non-linear) exhibits intensive non-linearity. (2) Adaptivity: a NN has the inherent ability to adjust the synaptic weights to adapt to the dynamics of the external environment, such as arbitrary pattern change. (3) Fault tolerance: when a part of a NN encounters a problem, the rest of the network can function, e.g., handling a segment contaminated with intensive interference. Our proposed design differs from that of Huang et al. (2017) in the following ways. (1) We removed the conventional and pooling layers because of non-stationary synchronous patterns, that is, different channels had various synchronization patterns that could be stronger or weaker. Convolution or pooling was likely to lose this kind of difference and could lead to loss of classification performance. (2) We set the merge layer to accept and merge all front layers to avoid the gradient vanishing of the front layers, while enhancing the weight propagation of the cross layers.

#### 3.2.3. Comparison with mutual information

To evaluate the improved MI based on AP clustering, we compared the classification performance between APCPMI and MI. The APCPMI between pairs of channels was calculated in terms of the default reference degree (see algorithm 1). After the EEG data of each channel was divided into five non-overlapping partitions with uniform distribution, the MI between bivariate signals was calculated. The reason for choosing five partition numbers was that a relatively higher standard deviation (enabling synchronization patterns to be more easily distinguished) was achieved when the signals were divided into five partitions (see Figure 3).

**Figure 8:**
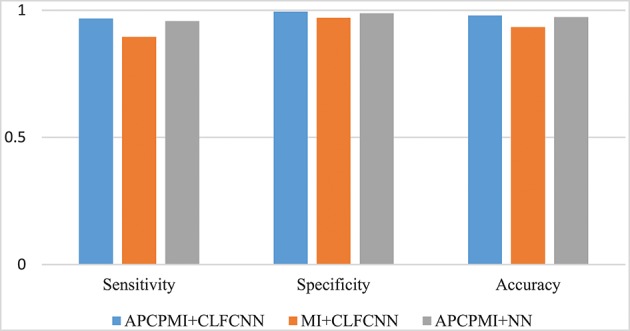
Comparison of the classification performance of APCPMI and MI. The Y-axis presents the accuracy (Equation 11). See text for details.

A comparison between the proposed approach and MI is provided in Figure 8. Straightforward classification accuracies were improved from 0.9335 to 0.9793, sensitivities from 0.8951 to 0.9676, and specificities from 0.9704 to 0.9942. Furthermore, a traditional NN, which was designed to remove connections between the cross layers, was also utilized to classify the APCPMIs. Straightforward performance was slightly improved, with an accuracy of 0.0065, sensitivity of 0.0108, and specificity of 0.006. In summary, we can conclude that the main improvement in classification was due to the APCPMI.

#### 3.2.4. Future work

It is believed that there exist global optimum reference degrees in the AP algorithm for special partitions. A suitable way to find the optimum reference degrees would be the deep reinforcement learning technique, which is a tree search algorithm based solely on reinforcement learning (Silver et al., 2017). This could improve the strength of the tree search, resulting in higher-quality performance in the next iteration, with potential applications in the field of game theory. These aspects were beyond the scope of the current study, but they could be further investigated in the future.

Epilepsy prediction aims to forecast seizures by differentiating between pre-seizure and post-seizure states in a dataset of intracranial EEG recordings. Seizure prediction is one of the most important areas of epilepsy research, and our work could be further developed in this direction.

The use of a single dataset means that the results should not be generalized to a wider population. In future work, multiple datasets will be created and used for validation of the method.

## 4. Conclusions

A lightweight cross-layer fully connected network was designed to adaptively describe the non-stationary synchronization pattern of epileptic seizures and to classify them effectively. In contrast to previous classifiers, we designed a merge layer to accept outputs from all previous connected layers and map them to the next layer one by one, so as to form feed-forward full connection blocks. This design could reduce gradient vanishing and enhance the transmission of cross-layer parameters.

Experimental results using scalp EEG data from the public dataset CHB-MIT showed that the proposed approach improved classification performance and achieved superior accuracy, specificity, sensitivity, Gmean, and FScore without losing the generalization capability of the classifier. Furthermore, the small standard deviations indicated the stability of the classifier.

Unlike other methods, this approach does not require any intentional preprocessing (removing noise, interference, and artifacts) to obtain higher classification performance. In addition, it only requires a hyper-parameter (time window), thereby avoiding the potential errors of the existing methods due to excessive parameter settings. In the case of a lack of *a priori* knowledge, this study has the potential to classify the complex synchronization patterns hidden in raw EEG data.

## Author contributions

FW and HK: contributed to the conception of the study. FW: conceived and designed the experiments. FW: Performed the experiments. HK: Analyzed the data. FW and HK: Contributed reagents, materials, and analysis tools.

### Conflict of interest statement

The authors declare that the research was conducted in the absence of any commercial or financial relationships that could be construed as a potential conflict of interest.

## Footnotes

^1^These quantities are not those used in classical signal detection theory (Macmillan and Creelman, 1991), despite the similarity in names.

## Supplementary material

Video S1 The learning curve of classifier to log accuracy and loss rates in the training stage.
